# Comparative Evaluation of the Effects of Sports Beverage and Whey Protein Concentrate on Surface Roughness and Microhardness of Nanofilled Composites: An In Vitro Study

**DOI:** 10.7759/cureus.114120

**Published:** 2026-08-07

**Authors:** Shayna U Shinde, Nilima Prakash, G. L Pradeep

**Affiliations:** 1 Dentistry, Mahatma Gandhi Vidyamandir’s Karmaveer Bhausaheb Hiray Dental College and Hospital, Nashik, IND; 2 Oral Pathology and Microbiology, Mahatma Gandhi Vidyamandir’s Karmaveer Bhausaheb Hiray Dental College and Hospital, Nashik, IND

**Keywords:** nanofilled composite, sports beverage, surface microhardness, surface roughness, whey protein

## Abstract

Background

Tooth-colored composite resins, especially nanofilled composites, have become the material of choice in restorative dentistry due to their excellent esthetics and durability. However, the increasing popularity of health supplements such as sports drinks and whey protein beverages raises concerns about their possible impact on the longevity of these restorations. Despite the wide use of these beverages, there is limited research regarding their effect on the surface properties of composite restorations. The purpose of this study was to assess and compare the effects of a commonly consumed sports beverage and whey protein concentrate on the surface roughness and microhardness of nanofilled composites.

Methodology

This in vitro study evaluated the effect of a popular sports drink and a whey protein concentrate on the surface roughness and microhardness of nanofilled composite resin. In total, 30 composite discs were prepared and divided into the following three groups (n = 10 each): Group A (immersed in whey protein concentrate), Group B (immersed in a sports beverage), and Group C (immersed in artificial saliva, control). Surface roughness was measured using a profilometer, and Vickers microhardness testing was performed at baseline and after repeated immersion for 30 days. Intragroup comparison was performed using the Wilcoxon signed-rank test (up to two observations). Intergroup comparison of the study group was performed using the Mann-Whitney U test. For all the statistical tests, a p-value <0.05 was considered to be statistically significant.

Results

Both the sports drink and whey protein groups showed a statistically significant increase in surface roughness and a decrease in microhardness compared to the control group. A greater rise in surface roughness was observed in discs immersed in the sports beverage, which was statistically highly significant (p = 0.005). However, a significant reduction in microhardness was observed following exposure to whey protein beverage (p = 0.005), indicating potential surface degradation of the composite material.

Conclusions

The use of acidic health supplements such as sports drinks and whey protein beverages deteriorates the surface characteristics of nanofilled composite restorations. There is a need to create awareness in those who consume such drinks frequently and advocate preventive care strategies.

## Introduction

Modern restorative dentistry emphasizes the vital role of esthetics and the minimally invasive concept. The increasing esthetic demands of patients have led to the popularity of tooth-colored restorative materials. Improvements in the formulation and simplification of adhesive procedures have enabled their use in the restoration of anterior and posterior teeth [1]. The modifications and improvements in composite materials do not compare with those in any other type of restorative material [2]. The size of filler particles has decreased continuously [3] to facilitate improved initial finish and gloss retention [4,5].

Nanocomposites incorporate nanosized particles into a matrix of a standard material and have been shown to possess superior mechanical properties and optical clarity compared with conventionally filled polymers [6]. There is evidence that nanofilled composite resins have superior surface characteristics and wear resistance comparable to hybrid composites, in addition to superior esthetics due to their high translucency and polishing ability. Nanofilled composites are used widely in clinical practice due to their longevity as restorations of anterior and posterior teeth [4].

Surface roughness and wear resistance are factors that influence the longevity of restorations [7]. Restorative materials remain vulnerable to surface changes from dietary habits and critical oral environment changes [8]. Increased surface roughness of restoration leads to plaque accumulation, staining, and possible gingivitis, thereby reducing the longevity of restorations [8,9]. This erosion of resin-based composite leads to anatomy loss, marginal discrepancy of restoration, and secondary caries [10]. Additionally, microhardness plays a pivotal role in the longevity of nanocomposites by influencing their resistance to deformation and wear, improving their performance in harsh environments.

The rising consumption of health supplements and sports/energy drinks among people of all age groups for sustaining physical fitness, driven by mass media and modern lifestyle, is a challenge [1,11]. Owing to their nutritional significance, whey proteins were added to this list of available supplements, causing a huge spike in their market size with a compound annual growth rate of 9.8% from 2019 to 2020. Traditionally, these supplements were consumed exclusively by athletes, but the inadequacies of modern diet and sedentary lifestyle have prompted the search for good sources of nutrition. Whey proteins are mostly available in the form of powders, which can be mixed in water and milk or as acidic beverages [11].

The intake of sports drinks has become increasingly common among young adults, particularly those engaged in physical activity, as they prefer them over carbonated beverages in recent years [12]. The acidic nature of these beverages may have detrimental effects on the properties of restorative resins [13]. Even the most advanced dental restorative materials remain susceptible to water sorption [14], insufficient polymerization, and humidity [15,16].

Although whey protein is considered a nutritional boon, it contains acidulants and carbohydrates (lactose), which are acidic and may pose a potential risk to teeth and restorative materials by altering the structure of composite resins [17-19]. Moreover, due to their viscous nature, they are in contact with the tooth and the restorative material for a longer time compared to other aerated drinks.

However, there is limited evidence available regarding the effect of sports drinks and whey protein supplements on restorative composite resin material. Their high consumption rate among younger patients, in those who engage in physical activity, and the growing use of resin-based restorative materials for this group necessitate the investigation of their effects on surface characteristics and wear resistance.

Hence, this study aimed to assess and compare the effects of a commonly consumed sports beverage and whey protein concentrate on the surface roughness and microhardness of nanofilled composites.

## Materials and methods

Study design

This in vitro comparative study was conducted at our Institute after obtaining ethical clearance from the Institutional Ethics Committee (approval number: MGV/KBHDC/1063/2023-24). This study involved the use of disc-shaped specimens made up of nanofilled composites divided into the following three groups: Group 1 comprised 10 discs of nanofilled composite resin immersed in whey protein concentrate, Group 2 comprised 10 discs of nanofilled composite resin immersed in a sports beverage, and the Control group comprised 10 discs of nanofilled composite resin immersed in artificial saliva. The study investigated the effect of immersion in a commonly consumed sports drink and whey protein concentrate on the surface roughness of nanofilled composite resin (FILTEK Z 350, 3M ESPE, Shade A2).

Disc preparation

**Figure 1:**
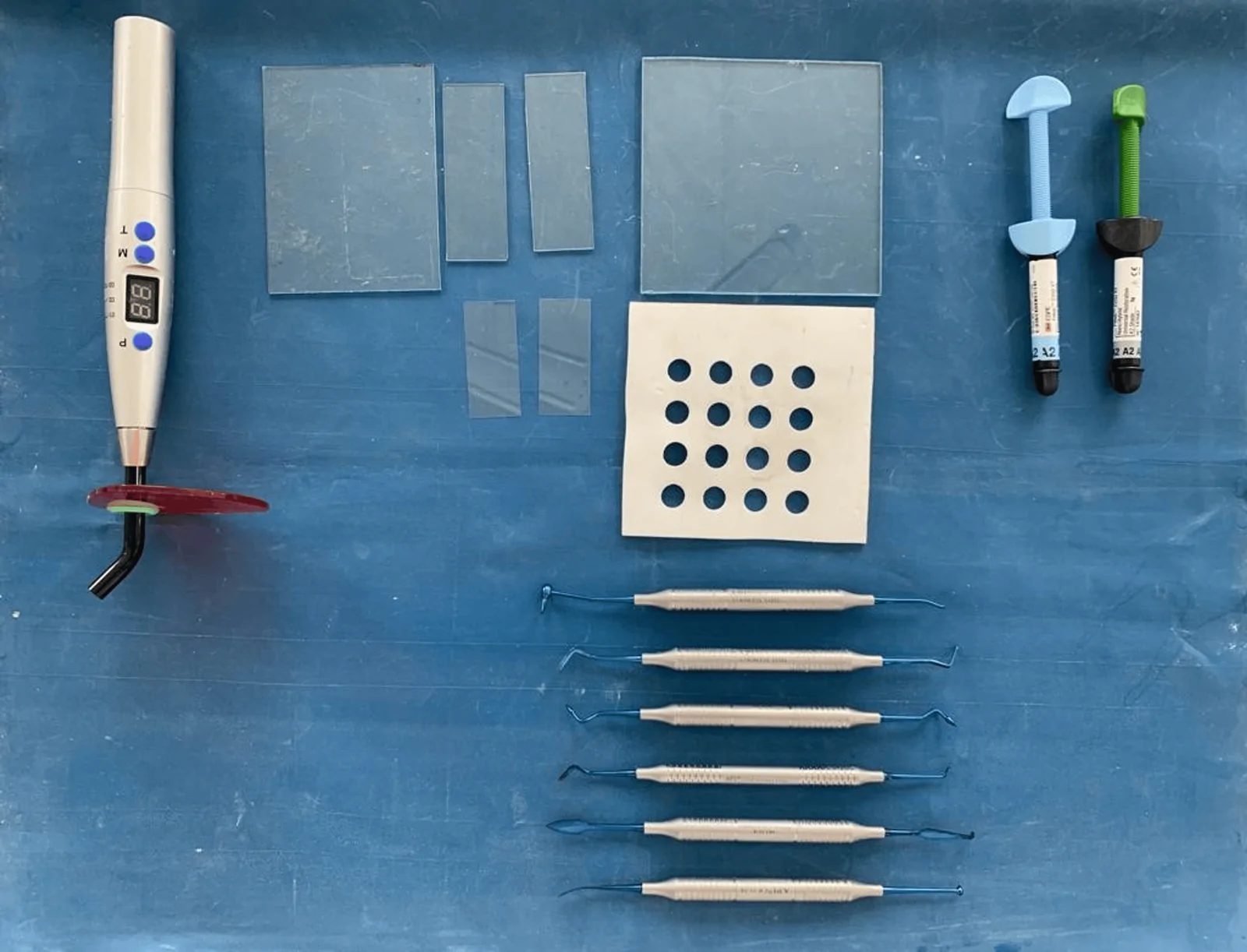
Armamentarium for the preparation of nanofilled composite resin disks.

In total, 30 samples were prepared using nanofilled composite resin using a Teflon mould of diameter 8 mm × 10 mm height (Figure 1). This aided in the standardization of the shape and size of each sample. Samples were made by filling a sufficient amount of material into the mould and pressing between mylar matrix strips supported by glass slides on either side. Only the mylar matrix strip and glass slide were used while making the samples before light polymerization. This helped in obtaining a smooth and flat surface. The discs were cured from both sides for 40 seconds with an LED curing unit operated in standard mode, with the guide tip placed perpendicular to the sample surface. The samples were placed at 37°C in a humid environment for 24 hours. This allowed post-irradiation hardening of composite restorations. Finishing and polishing were performed by wet-polishing in planar motion using coarse, medium, fine, and super fine abrasive discs, followed by ultrasonically cleaning in distilled water for 10 minutes [11].

Surface roughness

**Figure 2:**
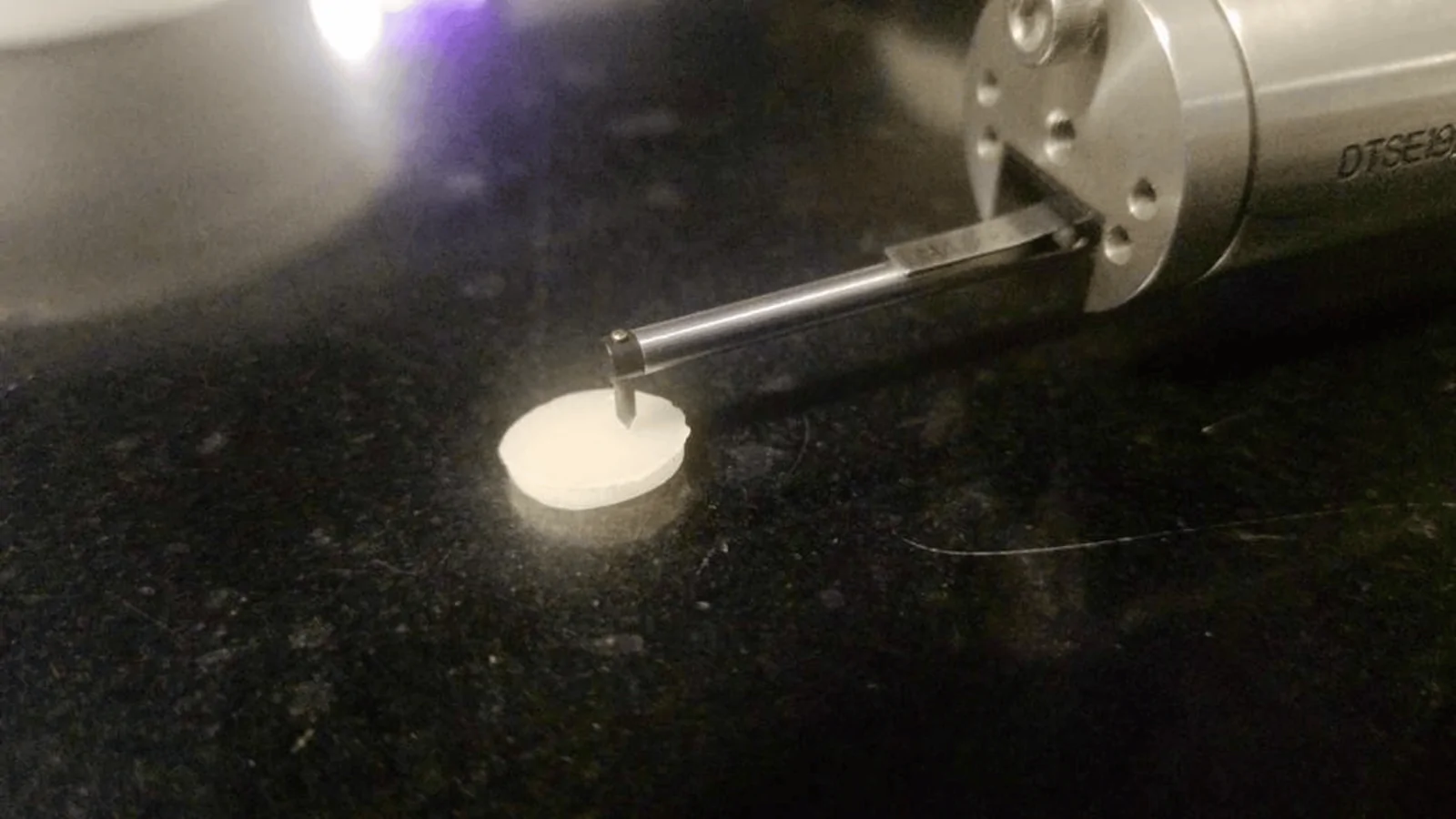
Surface roughness analysis of nanofilled composite resin disks using a Stylus profilometer.

Baseline surface roughness values were obtained for each sample using a stylus profilometer. The surface roughness was measured before and after immersion using a profilometer, with surface roughness (Ra) values calculated from three readings per sample. The points of roughness measurement were marked on the sample surface randomly. Measurements were performed at right angles to the samples (Figure 2).

Immersion protocol

**Figure 3:**
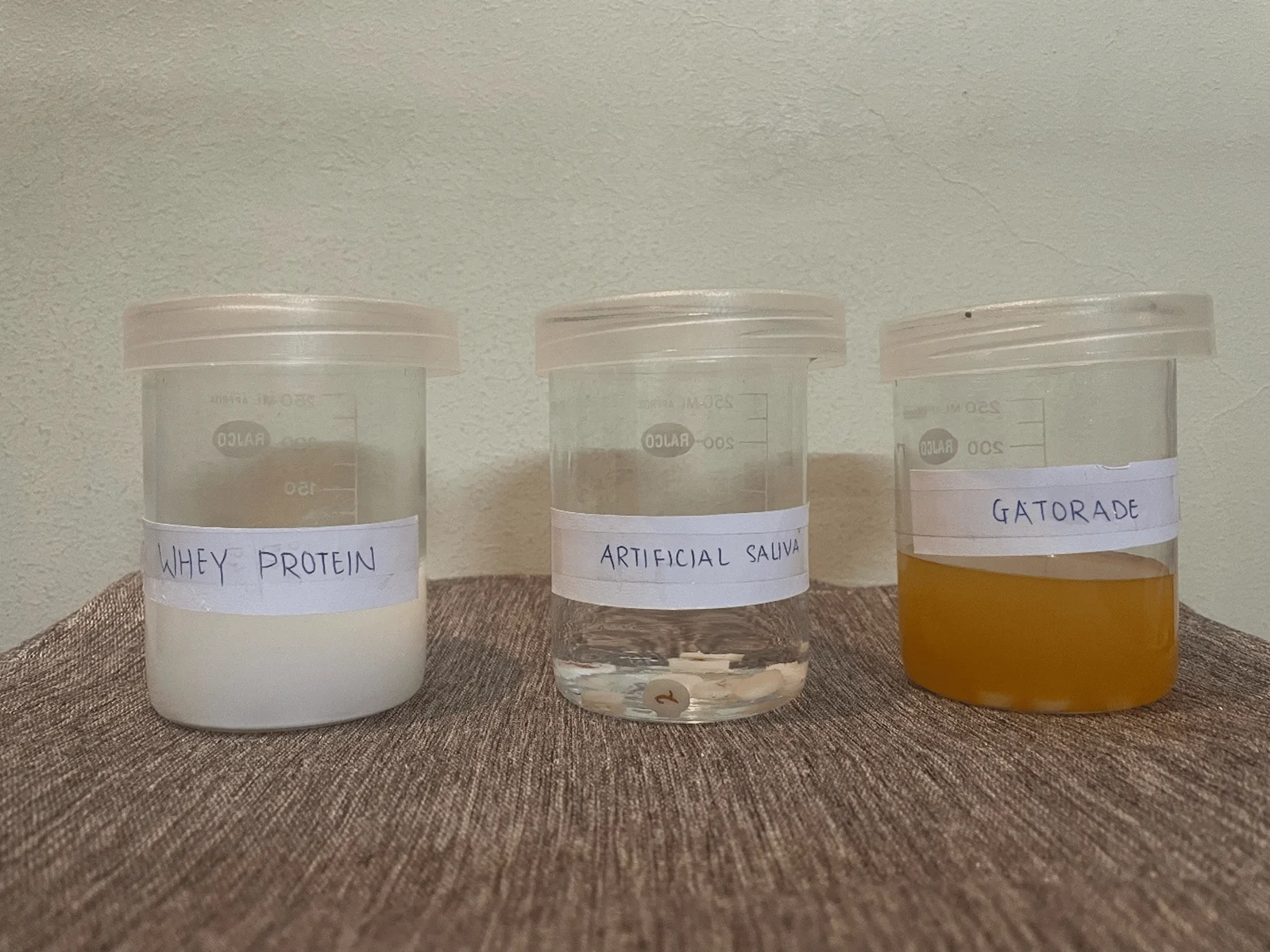
Nanofilled discs immersed in Whey protein concentrate (Group 1), sports beverage (Group 2), and artificial saliva (Control group).

**Figure 4:**
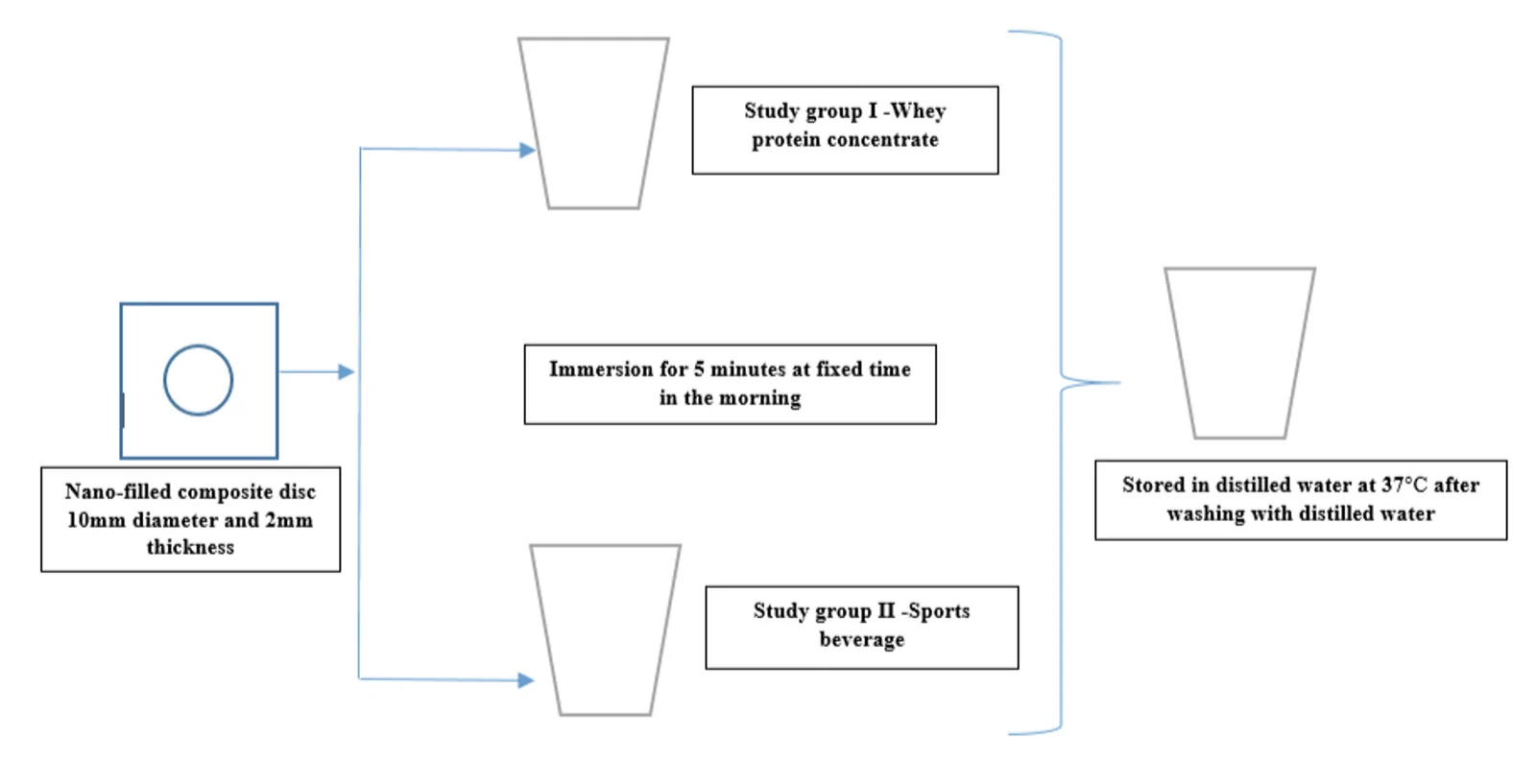
Steps in surface roughness and microhardness analysis of nanofilled composite resin disks post-immersion.

In the study, 10 discs each were immersed in whey protein concentrate (Group 1) and sports beverage (Group 2) for five minutes in the morning at a fixed time every day after obtaining baseline surface roughness and microhardness scores. After immersion in the solution, the samples were washed with distilled water and maintained in distilled water at 37°C during the rest of the day (Figures 3, 4). The beverage was replenished every day by using the same quantity for standardization. The containers were sealed to prevent evaporation of the test and control solutions. The cycle was repeated for 30 days [14]. In addition, 10 discs were placed in artificial saliva (Control group) for 30 days. Surface roughness values were obtained for each sample after immersion for 30 days using a profilometer.

Microhardness

**Figure 5:**
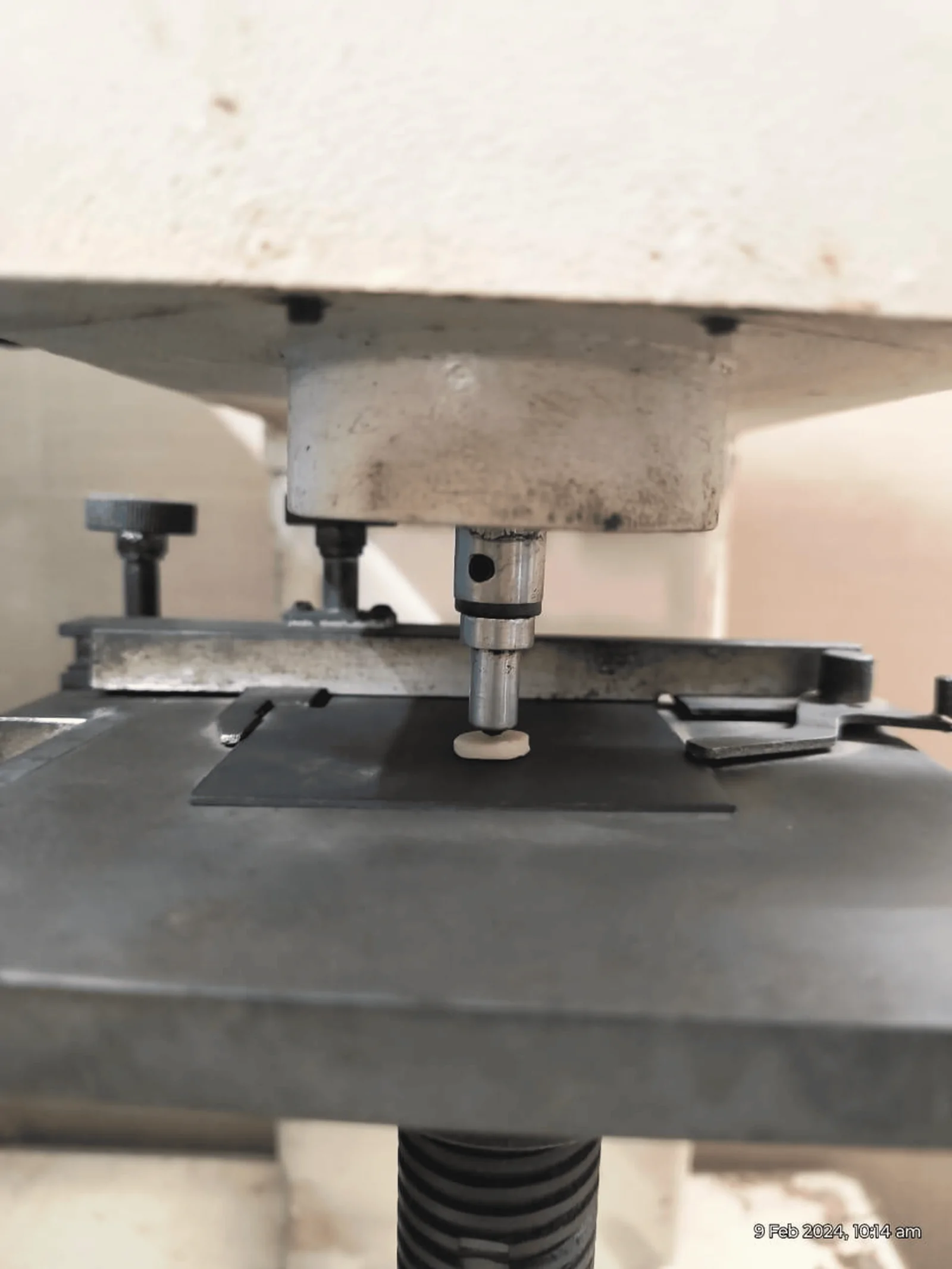
Microhardness tester for testing the microhardness of nanofilled composite disc.

Baseline microhardness evaluation was performed using a hardness tester (Figure 5). Three indentations were made, and measurements were obtained at different points on each specimen using a microhardness tester. Vickers hardness number (VHN) was obtained from the average. The measurements were taken at 1 mm from each other [11].

Control group

For the Control group, after recording the baseline surface roughness values and microhardness evaluation, the samples were immersed in artificial saliva for 30 days. Surface roughness and microhardness were assessed after 30 days.

Statistical analysis

Data obtained were compiled on an MS Office Excel Sheet version 2019 (Microsoft Corp., Redmond, WA, USA) and subjected to statistical analysis using SPSS version 26.0 (IBM Corp., Armonk, NY, USA). Descriptive statistics such as frequencies and percentages were calculated for categorical data. Mean and standard deviation were calculated for numerical data. The normality of numerical data was assessed using the Shapiro-Wilk test. Intragroup comparison was performed using the Wilcoxon Signed-rank test (up to two observations). Intergroup comparison of the study group was performed using the Mann-Whitney U Test. For all the statistical tests, a p-value <0.05 was considered to be statistically significant, keeping α error at 5% and β error at 20%, resulting in a study power of 80%.

## Results

The mean baseline score of surface roughness for nanofilled composite resin was found to be 0.15240. Following repeated immersion in whey protein concentrate (Group 1) for 30 days, the surface roughness increased to 0.208600 with a mean increase of 0.0562. This represents a ~36.9% increase in surface roughness. The difference in surface roughness was found to be statistically highly significant (p = 0.005). Nanofilled composite resin exhibited a higher surface roughness (0.23920) with a mean increase of 0.0868 following repeated immersion in a sports beverage (Group 2) for 30 days in comparison to the baseline score. This represents a ~57.0% increase in surface roughness. The difference in surface roughness was found to be highly statistically significant (p = 0.005). Artificial saliva (Control group) showed a negligible mean increase of only 0.0002 (reaching 0.15260), which was not statistically significant (p = 0.994) (Table 1).

**Table 1:** Comparison of surface roughness of nanofilled composite at baseline and after repeated immersion in whey protein concentrate (Group 1), sports beverage (Group 2), and artificial saliva (Control group) using Wilcoxon signed-rank test. **: statistically highly significant difference (p < 0.01); ^#^: non-significant difference (p > 0.05).

Groups	N	Mean values	Standard Deviation	Standard error mean	Mean difference	Z value	P-value
Study group 1							
Baseline	10	0.152400	0.0287719	0.0090985	0108240	-2.803	0.005**
Immersion in whey protein concentrate	10	0.208600	0.0342286	0.0108240			
Study group 2							
Baseline	10	0.152400	0.0287719	0.0090985	.0868000	-2.803	0.005**
Immersion in sports beverage	10	0.239200	0.0413543	0.0130774			
Control group							
Baseline	10	0.152400	0.0287719	0.0090985	-0.0002	-0.007	0.994^#^
Immersion in artificial saliva	10	0.152600	0.0290448	0.0091848			

On intergroup comparison of the mean surface roughness of nanofilled composite resin after immersion in whey protein concentrate (Group 1) and a sports beverage (Group 2), a statistically significant difference (p = 0.049) was noted with higher values for nanofilled composite resin disks immersed in the sports beverage (Group 2) (Table 2). Thus, the sports beverage was significantly more erosive than the whey protein concentrate.

**Table 2:** Intergroup comparison of post-immersion surface roughness among the study groups using Mann-Whitney U test. *: statistically significant difference (p < 0.05).

Group	N	Mean	Standard deviation	Mann-Whitney U value	Z value	P-value
Group 1 whey protein concentrate	10	0.208600	0.0342286	24.000	-1.965	0.049*
Group 2 sports beverage	10	0.239200	0.0413543			

The mean baseline score of microhardness for nanofilled composite resin was found to be 98.360. Following repeated immersion in whey protein concentrate (Group 1), the microhardness decreased to 87.5330 with a mean decrease of 10.8290. This represents an ~11.0% reduction in microhardness. The difference in microhardness was found to be statistically highly significant (p = 0.0001). The microhardness of nanofilled composite resin reduced to 90.5810 in Group 2 (sports beverage group) with a mean decrease of 7.7810 (dropping to 90.5810). This represents a ~7.9% reduction in microhardness. The difference in microhardness was found to be highly statistically significant (p = 0.005). Following immersion in artificial saliva, the nanofilled composite showed minimal reduction in microhardness (98.2660) in comparison to baseline scores. However, the difference was not statistically significant (p = 0.9592) (Table 3).

**Table 3:** Comparison of microhardness of nanofilled composite at baseline and after repeated immersion in whey protein concentrate (Group 1), sports beverage (Group 2), and artificial saliva (Control group) using Wilcoxon signed-rank test. **: statistically highly significant difference (p < 0.01); ^#^: non-significant difference (p > 0.05).

Groups	N	Mean values	Standard deviation	Standard error mean	Mean difference	Z value	P-value
Group 1							
Baseline	10	98.3620	4.19856	1.32770	10.82900	5.8002	0.0001**
Post-immersion	10	87.5330	4.15115	1.31271			
Group 2							
Baseline	10	98.3620	4.19856	1.32770	7.78100	-2.803	0.005**
Post-immersion	10	90.5810	3.87747	1.22616			
Control group							
Baseline	10	98.3620	4.19856	1.32770	0.09600	0.0511	0.9592^#^
Post-immersion	10	98.2660	4.19246	1.27099			

Intergroup comparison of the mean microhardness of nanofilled composite resin after immersion in whey protein concentrate (Group 1) and a sports beverage (Group 3) for 30 days was performed. Although whey protein caused a numerically greater reduction in structural hardness, the difference between the two study groups was not statistically significant (p = 0.059) (Table 4)

**Table 4:** Intergroup comparison of post-immersion microhardness among the study groups using Mann-Whitney U test. ^#^: non-significant difference (p > 0.05).

Group	N	Mean	Standard deviation	Mann-Whitney U value	Z value	P-value
Group 1	10	87.5330	4.15115	25.000	-1.891	0.059^#^
Group 2	10	90.5810	3.87747			

## Discussion

Resin-based composite materials are the most widely used restorative dental material due to their superior aesthetic properties and the ability to withstand high compressive forces [5]. The introduction of nanofilled composites marked a significant advancement in dental materials technology. These materials provide better polishing and aesthetic properties as nanoparticles have a much smaller size (typically less than 100 nm) compared to traditional filler particles, which allows for a closer packing of the particles within the resin matrix. They also provide wear resistance due to the high filler content required for posterior restorations [4].

Despite various advances, there is evidence that restorative materials are susceptible to surface changes. The onset of degradation of the restoration surface is dependent on the pH of the oral medium and the period of contact with the dietary solutions [2]. Consequently, in the short- or long-term, acidic solutions erode the agent coating the filler particles of the composite resin and modify its structure physically and chemically by altering the polymeric network [20,21]. Noteworthy among the surface characteristics are surface roughness and wear resistance, as they impact the longevity of restorations [20].

The purpose of the present study was to assess and compare the surface roughness and microhardness of nanofilled composite resin after repeated immersion in a popular sports beverage and whey protein concentrate.

The discs of nanofilled composite resin were evaluated for surface roughness and microhardness at baseline and following repeated immersion in a sports beverage and whey protein concentrate for 30 days using a Stylus profilometer and Vickers microhardness tester, respectively. During consumption of drinks, the drink contacts tooth surfaces and restorations for a short period, after which it is washed away by saliva. This in vitro study involved cyclic immersion of the discs in the study solutions to simulate the washing effect of saliva. The beverages were replenished every day during the study period to maintain their original pH level. The specimens’ immersion protocol simulated an individual consuming acidic food, sour fruits, and drinks (Figures 3, 4). After the cyclic immersion procedure was completed, the specimens were rinsed with distilled water, blotted dry [11], and subjected to testing for surface roughness and microhardness post-immersion. The values recorded were subjected to statistical analysis.

Surface roughness

The mean baseline score of surface roughness for nanofilled composite resin was found to be 0.152. Both whey protein concentrate (0.208) and sports beverages (0.239) significantly increased the surface roughness of nanofilled composites, with sports drinks causing the most substantial degradation. The difference in surface roughness was found to be statistically highly significant (p = 0.005). However, a marginal rise in the surface roughness (0.152) of the nanofilled composite was observed after immersion in artificial saliva for 30 days in comparison to baseline values, with no statistically significant results (p = 0.994) (Table 1). On intergroup comparison of the mean surface roughness of nanofilled composite resin, there was a statistically significant difference (p = 0.049), with higher values for nanofilled composite resin disks immersed in the sports beverage (Table 2). These findings align with research by Erdemir et al. [20], Vaidya et al. [1], and Hemalatha et al. [22], who reported that surface roughness of nanofilled composite resin increased progressively with immersion in acidic sports beverages.

Regarding whey protein concentrate, Santin et al. [18] reported an increase in the surface roughness of various composite resins following immersion in whey protein, depending on its chemical composition. Many sports drinks have a low pH, making them acidic. The acidic environment can lead to the erosion of the composite material, particularly affecting the resin matrix and leading to the exposure of filler particles. Acidic conditions can also cause hydrolytic degradation of the resin matrix in composite restorations. It is presumed that whey protein concentrate is less acidic; however, its viscous nature leads to longer contact time with the tooth and the restorative material, leading to chemical degradation of the resin matrix. As whey protein is mostly consumed post-exercise, the decreased salivary flow results in inadequate rinsing and buffering of demineralizing acids on tooth surfaces and restorations. This further increases their potential for erosion [8].

Microhardness

The mean baseline score of microhardness for nanofilled composite resin was found to be 98.360. A highly significant reduction in microhardness was observed in both study groups. A substantial reduction in microhardness was observed with whey protein concentrate (87.533) and, to a lesser extent, with sports beverage (90.581). The difference in microhardness was found to be statistically highly significant (p = 0.0001 and p = 0.005). However, the nanofilled composite discs showed minimal reduction in microhardness (98.2660) following immersion in artificial saliva with a statistically non-significant difference (p = 0.9592) (Table 3).

Kaur et al. [11] reported a decrease in microhardness of nanofilled composite resin following immersion in whey protein beverages. Few studies have investigated the role of whey protein use and its effects on nanofilled composite resin. Similarly, the reduction in microhardness from sports beverages is consistent with Erdemir et al. [21]. However, did not investigate the effects of sports drinks on nanofilled composite resins.

Microhardness is an extremely critical property of restorations as it directly affects the physicochemical properties such as compressive strength and abrasion resistance. Reduction in microhardness undermines the quality of restorations and accelerates the necessity of replacement [23].

It is presumed that the hygroscopic agents in whey protein concentrate, such as lactose, proteins, and other organic compounds, may interact with the resin matrix and filler particles, leading to an increase in water sorption. Additionally, the acidic pH of whey protein may erode the resin matrix. The surface degradation of restorative materials is also dependent on the filler and matrix composition of a specific material [11].

There is evidence that salivary flow rate reduces following whey protein beverage consumption [24]. This prevents the mechanical flushing and protection of teeth and restorative material by the cleansing properties of saliva. The longer contact with whey protein may facilitate increased reaction time and thus undermine the surface.

The reduction in microhardness following immersion in the sports beverage is due to the acidic pH that causes matrix decomposition, surface erosion, and dissolution. The acids present in these beverages penetrate the resin matrix and soften bisphenol A diglycidyl dimethacrylate (Bis-GMA) and facilitate the release of unreacted monomers. Urethane dimethacrylate, triethylene glycol dimethacrylate, and Bis-GMA are very susceptible to absorption and solubility, which may lead to softening and degradation of the resin matrix [23].

The results of the present study revealed alterations in the surface characteristics of nanofilled composite resins after exposure to sports beverage and whey protein concentrate with repeated immersion cycles for 30 days.

Study strengths

The strengths of the present study are a well-defined experimental design with standardized disc preparation and two outcome measures. Two clinically relevant beverages were included. The study involved a control group for better comparison.

Limitations and future prospects

The limitations include the small sample size, shorter duration of exposure time to the beverages tested, and the in vitro setting, which limited the generalizability of the results. To augment the current study’s findings about the degradation of composite resins subjected to dietary supplements, further in vitro and in vivo investigations that more closely mimic the oral environment are needed.

## Conclusions

In recent years, the intake of sports and energy drinks has increased among the adolescent population. Additionally, whey protein is used in infant formulas, sports bars, beverages, and powders as a dietary supplement due to its high nutritional composition. Composite resin restorations experience a wide variety of physical and chemical conditions within the oral environment, significantly affecting the breakdown of resin composites. In line with the results of the present in vitro study, we presume that the consumption of sports drinks and whey supplements may be detrimental to teeth and restorative materials. Further in vivo studies are recommended to determine clinical harm and long-term effects on patients, as an in vitro setting cannot fully replicate the complex, dynamic environment of the human mouth. Maintaining good oral hygiene practices can help mitigate the risks associated with acidic beverage consumption.

## References

[1] Vaidya N, Kumar P, Pathak K, Punia SK, Choudhary A, Patnana AK (2020). Comparative evaluation of the influence of different sports/energy drinks and alcoholic beverages on the surface roughness of three different flowable esthetic restorative materials: an in vitro analysis. J Int Soc Prev Community Dent.

[2] Camilotti V, Mendonça MJ, Dobrovolski M, Detogni AC, Ambrosano GM, De Goes MF (2020). Impact of dietary acids on the surface roughness and morphology of composite resins. J Oral Sci.

[3] Schmalz G, Hickel R, van Landuyt KL, Reichl FX (2017). Nanoparticles in dentistry. Dent Mater.

[4] Mitra SB, Wu D, Holmes BN (2003). An application of nanotechnology in advanced dental materials. J Am Dent Assoc.

[5] Kaizer MR, de Oliveira-Ogliari A, Cenci MS, Opdam NJ, Moraes RR (2014). Do nanofill or submicron composites show improved smoothness and gloss? A systematic review of in vitro studies. Dent Mater.

[6] Swift EJ (2005). Nanocomposites. J Esthet Restor Dent.

[7] Wu SS, Yap AU, Chelvan S, Tan ES (2005). Effect of prophylaxis regimens on surface roughness of glass ionomer cements. Oper Dent.

[8] Dunkin RT, Chambers DW (1983). Gingival response to class V composite resin restorations. J Am Dent Assoc.

[9] Chan KC, Fuller JL, Hormati AA (1980). The ability of foods to stain two composite resins. J Prosthet Dent.

[10] Yap AU, Wu SS, Chelvan S, Tan ES (2005). Effect of hygiene maintenance procedures on surface roughness of composite restoratives. Oper Dent.

[11] Kaur N, Nikhil V (2022). Do whey protein beverages affect the microhardness of composites? A laboratory study. J Conserv Dent.

[12] Coombes JS (2005). Sports drinks and dental erosion. Am J Dent.

[13] Nasim I, Neelakantan P, Sujeer R, Subbarao CV (2010). Color stability of microfilled, microhybrid and nanocomposite resins--an in vitro study. J Dent.

[14] Rahim TN, Mohamad D, Md Akil H, Ab Rahman I (2012). Water sorption characteristics of restorative dental composites immersed in acidic drinks. Dent Mater.

[15] Nedeljkovic I, Teughels W, De Munck J, Van Meerbeek B, Van Landuyt KL (2015). Is secondary caries with composites a material-based problem?. Dent Mater.

[16] de Paula AB, Fucio SB, Ambrosano GM, Alonso RC, Sardi JC, Puppin-Rontani RM (2011). Biodegradation and abrasive wear of nano restorative materials. Oper Dent.

[17] Beecher JW, Drake MA, Luck PJ, Foegeding EA (2008). Factors regulating astringency of whey protein beverages. J Dairy Sci.

[18] Santin DC, Naufel FS, Mondelli RF, Piccolotto A, Schmitt VL (2019). Effect of sports drinks on the surface properties of composite resins after prolonged exposure: in vitro study. Braz J Oral Sci.

[19] Cribb PJ, Hayes A (2006). Effects of supplement timing and resistance exercise on skeletal muscle hypertrophy. Med Sci Sports Exerc.

[20] Erdemir U, Yildiz E, Eren MM, Ozel S (2013). Surface hardness evaluation of different composite resin materials: influence of sports and energy drinks immersion after a short-term period. J Appl Oral Sci.

[21] Erdemir U, Yildiz E, Saygi G, Inan Altay N, Eren Mert M, Yucel T (2016). Effects of energy and sports drinks on tooth structures and restorative materials. World J Stomatol.

[22] Hemalatha Hemalatha, Nagar P (2018). A comparitive evaluation of the effect of sports and fruit drinks on the surface roughness of nanofilled composite and light cure GIC-an in vitro study. Int J Clin Pediatr Dent.

[23] Abouelmagd DM, Basheer RR (2022). Microhardness evaluation of microhybrid versus nanofilled resin composite after exposure to acidic drinks. J Int Soc Prev Community Dent.

[24] Norton V, Lignou S, Methven L (2021). Whey protein derived mouthdrying found to relate directly to retention post consumption but not to induced differences in salivary flow rate. Foods.

